# The quest for the best target genes for RNAi‐mediated pest control

**DOI:** 10.1111/imb.12966

**Published:** 2024-10-25

**Authors:** Doga Cedden, Gregor Bucher

**Affiliations:** ^1^ Department of Evolutionary Developmental Genetics, Johann‐Friedrich‐Blumenbach Institute, GZMB University of Göttingen Göttingen Germany

**Keywords:** dsRNA, pest management, RNA interference, target gene

## Abstract

RNA interference (RNAi) has emerged as an eco‐friendly alternative to classic pesticides for pest control. This review highlights the importance of identifying the best target genes for RNAi‐mediated pest control. We argue that the knowledge‐based approach to predicting effective targets is limited by our current gaps of knowledge, making unbiased screening a superior method for discovering the best target processes and genes. We emphasize the recent evidence that suggests targeting conserved basic cellular processes, such as protein degradation and translation, is more effective than targeting the classic pesticide target processes. We support these claims by comparing the efficacy of previously reported RNAi target genes and classic insecticide targets with data from our genome‐wide RNAi screen in the red flour beetle, *Tribolium castaneum*. Finally, we provide practical advice for identifying excellent target genes in other pests, where large‐scale RNAi screenings are typically challenging.

## Graphical abstract

- A major challenge for RNAi mediated pest control is the identification of the most effective RNAi target genes from the pool of ~37% of all genes that are essential.
- Effective RNAi target genes are predominantly highly conserved genes serving basic cellular functions such as protein degradation. Classic insecticide targets are mostly poor targets for RNAi‐mediated pest control.
- Several factors complicate the prediction of the most effective target genes such as protein stability. We provide suggestions on how to efficiently identify target genes based on unbiased screening efforts.

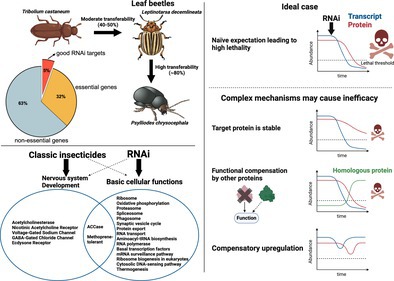

## INTRODUCTION

Currently, pest control strategies heavily rely on the application of classic pesticides. However, classic pesticides pose a great risk to non‐target organisms such as bees due to their non‐selective mode of action (Brittain & Potts, 2011; Lundin et al., 2015). In addition, pesticide‐resistant pest populations emerge due to the limited classic pesticide mode of action repertoire, leading to ineffective control in the field (Chen et al., 2023; Willis et al., 2020). It is becoming ever more challenging to discover and register classic pesticides with novel modes of action because of the complexity of the protein‐pesticide interactions and the required environmental safety (Umetsu & Shirai, 2020). Hence, alternative strategies must be developed for sustainable pest control. RNA interference (RNAi) has emerged as an eco‐friendly alternative to classic pesticides (Baum et al., 2007; Mao et al., 2007). RNAi‐based pest control strategies include the delivery of double‐stranded RNA (dsRNA) through exogenous spray applications or cultivating dsRNA‐transcribing transgenic crops. Upon feeding, the dsRNAs are taken up by the pests and enter cells. There, they are processed by the RNAi machinery into small interfering RNAs (siRNA) and guide the cleavage of complementary mRNA (Tijsterman & Plasterk, 2004). Many but not all insects effectively take up dietary dsRNAs into the cytoplasm. For instance, many coleopterans, especially leaf beetles, can be targeted quite well while many lepidopterans cannot (Shukla et al., 2016; Willow & Veromann, 2021). Although virtually any gene can be targeted by exploiting the RNAi pathway, not all genes are equally suitable for pest control.

**FIGURE 1:**
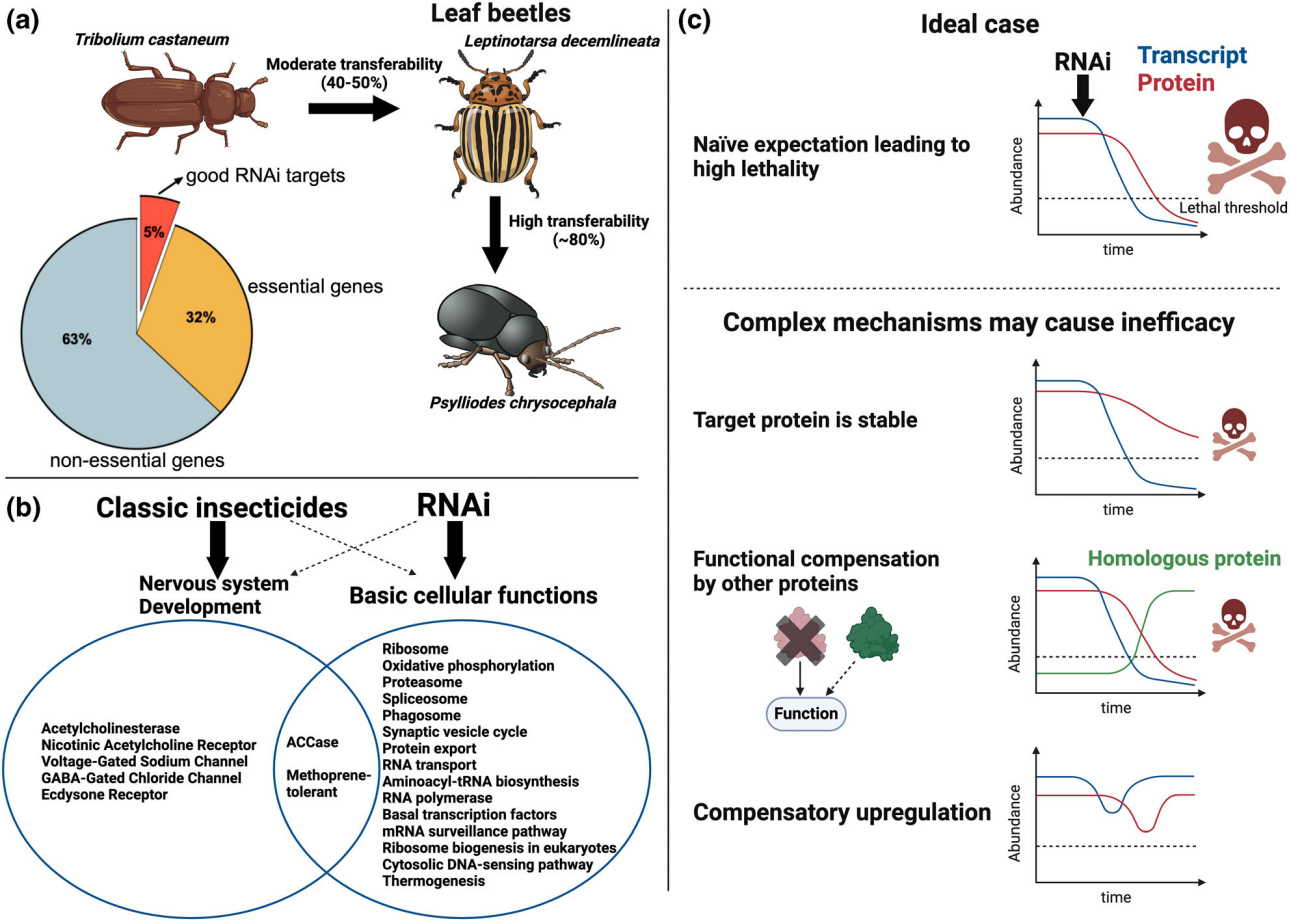
Overview of the best target genes for RNAi mediated pest control. (a) An injection‐based genome‐wide RNAi screen in *Tribolium castaneum* identified good target genes (above 90% mortality), which is a subset of essential genes (37%). The transferability was moderate (40%–50%) when the most effective target genes from *T. castaneum* were tested in another leaf beetle through a different delivery (oral application). In contrast, the transferability was high (80%–90%) when targets that already worked in one leaf beetle by oral uptake were tested in another leaf beetle. (b) Effective RNAi target genes are related to basic cellular functions such as translation and proteasome‐mediated protein degradation rather than to genes whose protein products are targeted by the most popular classic insecticides, which mainly target the nervous system. Nonetheless, some classic insecticide targets, such as acetyl‐CoA carboxylase (ACCase), were also effective RNAi targets, suggesting the presence of a minor overlap between the targets of the two pest control strategies. (c) In the ideal case, RNAi depletes the target transcript followed by depletion of the protein product due to natural protein turnover. Decrease of the protein level below a crucial threshold leads to lethality. However, there are several factors that may counteract this desired effect. If the target protein is very stable, its levels are depleted much more slowly despite transcript degradation. Moreover, the function of a target protein can be rescued by related proteins, that might even be upregulated by the cell in order to compensate for the loss. Further, the transcription of the target gene itself might be upregulated by a regulatory gene network in response to RNAi mediated knock‐down. This compensatory transcription will reduce the degree of knock‐down that can be achieved. These and other potential issues add several layers of complexity to the prediction of target genes. Given that most of these parameters are unknown for most genes, unbiased screen‐based approaches have an advantage in finding effective RNAi target genes because they use lethality as criterion for selection (see Table 2).

A key step for the establishment of RNAi in the control of a certain pest is the identification of a target gene. This should be an essential gene, i.e. a gene the knockdown of which leads to the death of the animal. Actually, more than a third of all insect genes lead to death when mutated or knocked down. The portion was estimated to be around 37% based on a systematic mutation screen in the vinegar fly *Drosophila melanogaster* and a large‐scale RNAi screen in the red flour beetle *Tribolium castaneum* (Figure 1a) (Mullins et al., 1994; Nusslein‐Volhard, 1994; Schmitt‐Engel et al., 2015; Wieschaus et al., 1984). Hence, it is actually not difficult to find an essential gene. The true challenge is the identification of those essential genes that are the most effective RNAi target genes.

Different strategies have been applied to identify target genes. Some efforts were inspired by the mode of action of classic pesticides, which mostly target the nervous system of arthropods. The underlying assumption was that the respective targets would be good targets for RNAi as well. For instance, the RNAi‐mediated knockdown of the voltage‐gated sodium channel, which is the target of the pyrethroid insecticides, caused up to 65% mortality in the peach‐potato aphid *Myzus persicae* (Tariq et al., 2019). Similarly, the mode of action of organophosphate insecticides was mimicked via the RNAi‐mediated knockdown of the acetylcholinesterase, causing mortality in the whitefly *Bemisia tabaci* (Malik et al., 2016). In a second strategy, knowledge about biological processes and their putative core genes has been used to select the targets. For instance, in one of the seminal papers on RNAi in pest control, Mao et al. (2007) used the known metabolite detoxification pathway of the cotton bollworm (*Helicoverpa armigera*) to devise a knowledge‐based RNAi strategy. Specifically, the respective detoxification gene was targeted by RNAi, leading to reduced resistance of the pest to the plant metabolite and subsequent reduced growth of cotton bollworm larvae (Mao et al., 2007). However, to our knowledge, these strategies have not resulted in commercial RNAi‐based products or are currently being considered for registration. The third approach combined small or mid‐scale screens with previous knowledge to select targets. For instance, in the second seminal paper on RNAi in pest control, Baum et al. selected 290 putatively essential genes based on previous knowledge and tested them in an RNAi screen to identify the best targets for control of the western corn rootworm *Diabrotica virgifera virgifera* (Baum et al., 2007). This approach led to the identification of target genes that showed response in other pest species as well and they have been used in a current product (SmartStax® PRO, patent WO2021216571A1), where transgenic corn expresses the dsRNA of one of the identified target genes (Baum et al., 2007; Bolognesi et al., 2012).

Finally, a large‐scale unbiased screening approach was used, where almost all protein coding genes from the red flour beetle *Tribolium castaneum* were screened without prior selection and the target genes were identified exclusively based on their efficacy in killing the pest. As this approach was not restricted by previous knowledge, it has allowed the identification of unexpected target pathways. For instance, Ulrich et al. found the proteasome as one of the best targets in the *T. castaneum* (Ulrich et al., 2015) and indeed, one of the first commercial sprayable dsRNA pesticides is targeting a proteasome component (Rodrigues et al., 2021). Recently, that screen was completed such that a comprehensive view on the RNAi‐induced lethality is now available for almost the entire proteome of a beetle (Buer et al., 2024).

In this paper, we emphasize the importance of identifying the best target genes for RNAi mediated pest control, considering that the different mode of action of RNAi is likely to work best with different subsets of target genes compared to classic pesticides. We argue that the knowledge‐based approach of predicting good targets is hampered by our lack of knowledge and that unbiased screening is a more powerful way to gain comprehensive insight into the best target processes and genes. In this review, we will expand on these issues and provide evidence by comparing the efficacy of previously suggested target genes with data from our genome wide dataset. Finally, based on our current knowledge, we propose an efficient route to find excellent target genes for other pests where large‐scale RNAi screenings are usually challenging.

## OPTIMIZING TARGET GENES MATTERS

The potential of species‐specific and eco‐friendly pest control using RNAi is hampered by some issues, which call for selecting the best possible target genes. For instance, the strength of the RNAi effect after feeding is reduced in many species by dsRNA degradation either in the gut or the hemolymph (Garbutt et al., 2013; Kunte et al., 2020; Peng et al., 2020; Wynant et al., 2014), potentially also by varying RNAi efficiency in populations (Mehlhorn et al., 2020), and by less efficient cellular uptake, systemic spread or intracellular transport of dsRNAs (Miller et al., 2008; Shukla et al., 2016; Tassetto et al., 2017; Yoon et al., 2017). Further, the incubation time between oral uptake of dsRNA and death of the pest is much longer than for classic pesticides—death is rarely observed before a week of incubation (Baum et al., 2007; Ulrich et al., 2015; Willow et al., 2021), and even incubation times more than 10 days have been described for some treatments (Cedden et al., 2024). Even considering that feeding may cease well before the death of an animal (Baum et al., 2007; Mehlhorn et al., 2021; Rodrigues et al., 2021), the pests will continue damaging the crops for much more time compared to classic pesticides, which can paralyse arthropods within hours post‐exposure (Narahashi, 1976; Wickham et al., 1974). Moreover, the moderate chemical stability of the dsRNA biomolecule leads to comparably quick degradation in the environment, quickly reducing the dsRNA availability in the field within a week (Dubelman et al., 2014; Parker et al., 2019). Finally, reduction of production costs is key when considering spraying dsRNA in broadacre applications—even a minor reduction of the required amount of dsRNA per hectare will increase revenue. In summary, finding none less than the best target genes is crucial to make sure that pests die as quickly as possible after the minimal amount of oral dsRNA uptake.

## CLASSIC TARGETS FOR A NON‐CLASSIC MODE OF ACTION?

The mode of action of RNAi is quite different from the mode of action of classic pesticides. For instance, many classic pesticides (e.g. contact insecticides) penetrate the cuticle and readily pass through cell membranes due to their small and lipophilic structures (Gerolt, 1983), and they usually act on an accessible site of a specific target protein. Targeting extracellular domains of proteins is possible and may even be advantageous because intracellular detoxification is circumvented. dsRNA molecules, in contrast, are comparably large molecules that need to be taken up via the gut, which is a challenging environment for biomolecules. Furthermore, dsRNA needs to be actively imported across the cell membrane (Saleh et al., 2006) because RNAi acts only intracellularly. On the other hand, the mRNA of a target gene can be degraded by RNAi irrespective of the location and accessibility of the protein produced by that target mRNA. Hence, RNAi is capable of causing lethal effects via the knocking down of genes encoding for extracellular or receptor proteins (Kola et al., 2019; Yu et al., 2014) as well as intracellular proteins or targets that are part of protein complexes including ribosomal proteins and proteasome subunits (Rodrigues et al., 2021; Ulrich et al., 2015).

Several reasons explain this discrepancy between RNAi and classic insecticides. First, classic insecticides must physically contact their target proteins, which might be inaccessible if they are part of complex structures. For instance, a popular insecticide group, organophosphates, inhibits the acetylcholinesterase in the synaptic cleft (Tsai & Lein, 2021), and yet another common insecticide group, pyrethroids, targets the accessible sites in the voltage‐gated sodium channels. Basic intracellular proteins such as ribosomes have not become targets of classic insecticides. In contrast, mRNAs of all types of proteins can be degraded in the cytoplasm by the RNAi machinery. Second, a subset of potential protein target sites for chemical insecticides might be highly conserved across the animal kingdom, posing a great risk to other organisms and restricting their commercialization (Casida, 2009). In contrast, RNAi is applicable even to target proteins that are highly conserved between pests and beneficials or even humans, because nucleic acid sequences are usually diverged enough to design taxon‐specific dsRNA (Castellanos et al., 2022; Cedden et al., 2025; Chen et al., 2021; Good et al., 2016). Hence, proteins can be targeted by RNAi that might not be targetable by classic pesticides due to safety issues. Given all these differences in size, delivery, and mode of action, it is rather expected that the optimal target genes for RNAi‐mediated pest control be quite different from those of classic pesticides.

The comparison provided in Table 1 suggested that the protein targets of the commonly used five classic insecticide groups were not among the most effective RNAi target genes (Clusters 1–3 in Buer et al., 2024 exhibiting optimal dsRNA dose–response) identified in the genome‐wide RNAi screen in *T. castaneum* (Table 1). The most common classic insecticide groups target nervous system‐associated proteins, while we found that the most effective RNAi target genes were related to basic cellular functions including translation, oxidative phosphorylation, and protein degradation. More specifically, the larval mortality after RNAi targeting classic insecticide targets resulted in low to moderate mortality (10%–50%) in *T. castaneum*, while the same screen identified 905 target genes with 90%–100% larval mortality. Apparently, there is not much overlap between effective RNAi target genes and common classic insecticide targets (Figure 1b).

**TABLE 1:** Comparison between classic insecticide and RNAi targets.

Classic insecticide group	Target	Larval mortality after RNAi^a^	Included in most effective RNAi target genes^a^
Organophosphates and Carbamates	Acetylcholinesterase	30%	No
Neonicotinoids	Nicotinic acetylcholine receptor^a^	50%	No
Pyrethroids	Voltage‐gated sodium channel^b^	10%	No
Organochlorines	Gaba‐gated chloride channel	22%	No
Juvenile hormone analogues	Methoprene‐tolerant^b^	100%	No
Diacylhydrazines	Ecdysone receptor	40%	No
Tetronic and tetramic acid derivatives	Acetyl‐CoA carboxylase	100%	Yes

^a^ Based on a genome screen in the red flour beetle *T. castaneum* Buer et al. (2024).

^b^ There are multiple subunits/paralogs for these targets (according to ibeetle‐base.uni‐goettingen.de). For this comparison, the subunit associated with the highest mortality in the primary screen was taken as the reference.

We note that the nervous system does not seem to be a highly effective RNAi target based on the genome wide RNAi screen in *T. castaneum* (Table 1) and no nervous system targeting RNAi‐based insecticide has been developed into a commercial product so far. The different modes of action readily explain that finding: Classic insecticides modulate the actions of their target proteins directly, e.g. by inhibiting the closure of sodium channels, to cause immediate effects (Soderlund, 2012), whereas the mode of action of RNAi is the reduction of protein levels. Effectively targeting key molecules of the nervous system is well possible by extracellular small molecules and leads to a fast response, while an RNAi mediated response requires the dsRNA to be taken up orally, to pass the gut and travel via the hemolymph to neural cells, which need to take the dsRNA up before mounting the depletion of corresponding mRNAs and consequently of the protein products. It is therefore not unexpected that other cell types may be more readily targeted by RNAi. It should be also noted that nervous system related targets might be still effective against non‐coleopteran pests such as mosquitoes (see Hapairai et al., 2017).

We also included in our comparison three non‐nervous‐system‐specific classic insecticides that are less popular but still commercially used. Several juvenile hormone receptor modulators such as methoprene are used as classic insecticides (Wilson, 2004) and RNAi against a receptor of the juvenile hormone, namely methoprene‐tolerant (Konopova & Jindra, 2008), caused 100% larval mortality. While being a good target (>90% larval mortality at 300 ng/μL dsRNA treatment), this gene was not included in the list of our most effective RNAi target genes, which was defined by 100% larval mortality at 30 ng/μL dsRNA treatment in Buer et al. (2024). Interestingly, Acetyl‐CoA carboxylase was included in the most effective RNAi target genes and that enzyme is targeted by the insecticide group containing tetronic and tetramic acid derivatives (Lümmen et al., 2014). Hence, there is a minor degree of overlap between the targets of classic insecticides and effective RNAi target genes but only Acetyl‐CoA is an excellent target for both modes of action.

## INSUFFICIENT KNOWLEDGE FOR THE KNOWLEDGE‐BASED APPROACH?

Another strategy to identify target genes has been the knowledge‐based approach. Essentially, target genes are selected for testing based on literature from model systems that indicate the essentiality of those genes. Indeed, this approach led to the identification of essential genes in pest species with high likelihood. For instance, the ecdysone receptor is an essential gene in *Drosophila melanogaster*, and its RNAi‐mediated knockdown leads to mortality in several pests (Yan et al., 2016; Yu et al., 2014). However, the fact that an RNAi knockdown of a gene leads to lethality does not yet make it a very good target, given that around 37% of all genes fit that criterion (see above, Figure 1a). Rather, we believe that our current knowledge is not comprehensive enough to reliably predict the best target genes, i.e. those that lead to death most rapidly with the lowest amounts of dsRNA. Our argument rests on the fact that we continue to ignore many of the key parameters of gene expression dynamics and gene function, which have profound effects on the efficiency of an RNAi‐mediated knockdown (Figure 1c).

First, the accessibility of cells or organs to dsRNA may differ such that some theoretically excellent target genes may be ‘protected’ by their cellular environment. For instance, RNAi targeting an essential gene that is expressed in the brain requires the passage of dsRNA through the gut, hemolymph and in contrast to other organs also the blood–brain barrier (Carlson et al., 2000; Zhang et al., 2022). In contrast, essential genes expressed in gut tissue are much more directly accessible because gut cells are the first point of contact with orally uptaken dsRNA and they are actively taking up substances from the gut juice. Likewise, hemocytes with their propensity of taking up substances from their environment may be more amenable than other cells. For instance, dsRNA uptake has been shown for *D. melanogaster* hemocytes but seems to be limited or non‐existent in other cells (Miller et al., 2008). Second, high protein stability will lead to enduring gene function even if the mRNA is efficiently destroyed (Figure 1c). Third, expression dynamics are likely to influence the effect of a knockdown. For instance, lowly expressed genes with short mRNA half‐life may be harder to knockdown compared to highly expressed genes with stable mRNA (Chen et al., 2021). Fourth, gene regulatory feedback loops may lead to a compensatory upregulation of transcription after knockdown thereby dampening the effect (Figure 1c). For instance, after knocking down the *Tc‐foxQ2* gene in the red flour beetle, some expression domains were knocked‐down more efficiently than others, probably reflecting different regulatory dynamics (Kitzmann et al., 2017). Fifth, related genes (e.g. paralogs) or pathways may be able to take over parts of the function of a knocked‐down gene (Figure 1c) thereby reducing lethality (Chen et al., 2012; Kitzmann et al., 2013). Sixth, the available data on gene function stems mainly from work in very few model systems (*D. melanogaster*, *Caenorhabditis elegans*, *Danio rerio*, *Mus musculus*, *Saccharomyces cerevisiae*), reducing the search for candidate genes to the biology of those organisms. Finally, much of the knowledge on gene function has been gathered by mutant analyses in model systems while reverse genetics knockdown may result in different dynamics of gene function loss and in consequence to modulated phenotypes (Kok et al., 2015). These and other parameters may strongly affect the efficacy of RNAi‐mediated pest control, but they remain unknown for most genes of most organisms, rendering predictions of target gene efficacy difficult at best.

The genome‐wide dataset gathered in the iBeetle‐screen allowed for scoring the efficacy of published target genes with our unbiased approach in the red flour beetle (Buer et al., 2024). For several common target genes in the literature, we asked in Table 2, by which approach they had been identified and how well they performed in the screen in the beetle (Table 2). We distinguished three main approaches, namely, purely knowledge‐based and focused on one or a few target genes (e.g. Kumar et al., 2009; Pridgeon et al., 2008; Tian et al., 2009; Vélez et al., 2019; Yu et al., 2014; Zhu et al., 2011), semi‐knowledge‐based mid‐scale screens (e.g. Baum et al., 2007) and unbiased large‐scale screens (e.g. Buer et al., 2024; Ulrich et al., 2015). We observed that only 33% (2 out of 6) of the targets identified through knowledge‐based selection were included in the good or most effective target genes in the genome‐wide screen in *T. castaneum* (Table 2). In contrast, 66% (4 out of 6) of the targets identified by mid‐scale screening approach were included in the good or most effective target genes in the genome‐wide screen, respectively. In line with that, both target gene families used in the two currently registered products *Snf7* (SmartStax®PRO transgenic maize) and *Proteasome Subunit Beta Type‐5* (Calantha™ sprayable dsRNA) were first identified through mid‐scale (Baum et al., 2007) or by unbiased screening (Ulrich et al., 2015) screens, respectively. This analysis suggests that screen‐based approaches might be more suitable for identifying effective RNAi target genes with commercialization potential compared to pure knowledge‐based approaches. The limitation of this investigation was that a genome‐wide RNAi screen data is only available for *T. castaneum*, making it challenging to generalize our statement across all pest species. For instance, target genes such as chitin synthase and ecdysone receptor investigated in Table 2 may be considered effective targets in some species other than *T. castaneum*, such as in the brown planthopper *Nilaparvata lugens* (Yu et al., 2014) and grain aphid *Sitobion avenae* (Yan et al., 2016). A direct comparison in these species would be interesting to see, in how far there could be exceptions to our conclusion that screen‐based approaches are superior to knowledge‐based approaches.

**TABLE 2:** Comparison between common RNAi target genes and the genome‐wide screen in *Tribolium castaneum* (Buer et al., 2024).

Target gene		Species and reference	Good target gene (5.8% of all genes)^a^	Most effective target genes (0.9% of all genes)^a^	Approach used in first identification^b^
Registered products					
*Snf7* (SmartStax®PRO)		*D. virgifera virgifera* (Baum et al., 2007)	Yes	Yes	Mid‐scale knowledge‐based screen (Baum et al., 2007)
*Proteasome subunit beta type‐5* (Calantha™)		*L. decemlineata* (Rodrigues et al., 2021)	Yes	No	First detected by an unbiased screen (Ulrich et al., 2015)
Common targets
*V‐ATPase* subunits		*D. virgifera virgifera* (Baum et al., 2007)	Yes	Yes	Mid‐scale knowledge‐based screen (Baum et al., 2007)
*α‐Tubulin*		*D. virgifera virgifera* (Baum et al., 2007)	No	No	Mid‐scale knowledge‐based screen (Baum et al., 2007)
*β‐Actin*		*L. decemlineata* (Baum et al., 2007)	No	No	Mid‐scale knowledge‐based screen (Baum et al., 2007)
*Smooth septate junction* (*SSJ*)		*D. virgifera virgifera* (Hu et al., 2016)	Yes	Yes	Knowledge‐based target (Hu et al., 2016)
*Heat shock protein 70*		*Agrilus planipennis* (Rodrigues et al., 2018)	Yes	Yes	First detected by an unbiased screen (Ulrich et al., 2015)
*Sec23*		*L. decemlineata* (Zhu et al., 2011)	Yes	Yes	Knowledge‐based target (Vélez et al., 2019; Zhu et al., 2011)
*Inhibitors of apoptosis*		*Aedes aegypt*i (Pridgeon et al., 2008)	No	No	Knowledge‐based target (Pridgeon et al., 2008)
*COPI coatomer β subunit*		*D. virgifera virgifera* (Baum et al., 2007)	Yes	Yes	Mid‐scale knowledge‐based screen (Baum et al., 2007)
*Ribosomal protein L19*		*D. virgifera virgifera* (Baum et al., 2007)	Yes	No	Mid‐scale knowledge‐based screen (Baum et al., 2007)
*Chitin synthase*		*Spodoptera exigua* (Tian et al., 2009)	No	No	Knowledge‐based target (Tian et al., 2009)
*Acetylcholinesterase*		*Helicoverpa armigera* (Kumar et al., 2009)	No	No	Knowledge‐based target (Kumar et al., 2009)
*Ecdysone receptor*		*Nilaparvata lugens* (Yu et al., 2014)	No	No	Knowledge‐based target (Yu et al., 2014)

^a^ Based on a genome screen in the red flour beetle *T. castaneum* (Buer et al., 2024).

^b^ The list was curated based on the first publication in which the target was identified as effective. However, it is possible that proprietary data produced by companies or unpublished studies might have identified the same target through a different approach prior to the publications listed here.

## LARGE‐SCALE SCREENING: UNBIASED BRUTE FORCE

Given the above arguments, we argue that many excellent target genes may not have been included in knowledge‐based approaches and our view on the best target processes may be incomplete due to the bias introduced by selecting the genes to be tested. A more unbiased screening approach has the potential to overcome these issues and generate a more comprehensive view. Essentially, the potentially biasing step of selecting candidate genes is avoided by screening many randomly selected or all genes. While a genome wide unbiased screen may not be the most intellectually challenging approach, it has proven a valuable (and Nobel Prize winning) key resource in the fields where understanding the function of genes is the aim (Boutros & Ahringer, 2008; Jorgensen & Mango, 2002; Nusslein‐Volhard, 1994; Patton & Zon, 2001; St Johnston, 2002).

Such a high‐throughput approach requires a robust experimental system, where animals are available in large amounts all year round and the application of dsRNA can be scaled up. We used the red flour beetle *T. castaneum* for that purpose for two reasons: First, it has a very strong and robust RNAi response, where injection of dsRNA into embryos or the hemolymph leads to knockdown in all cells and even the offspring of injected females (Brown et al., 1999; Bucher et al., 2002; Miller et al., 2008; Tomoyasu & Denell, 2004). Further, a number of transgenic and genome editing techniques have been established, opening the potential for in depth follow‐up studies (Klingler & Bucher, 2022). Besides a screen aimed at pondering gene function with respect to developmental and physiological processes (Hakeemi et al., 2022; Schmitt‐Engel et al., 2015), we performed another genome wide screen to specifically ask for lethality after larval injection (Buer et al., 2024). In that screen, we aimed at testing all protein coding genes without prior selection. Hence, it was unbiased with respect to protein coding genes, but it was biased in that it did not include any other target class such as non‐coding RNAs. In a primary high‐throughput screen, 15,530 genes were screened by injecting a comparably high concentration of dsRNA into larvae and scoring for their death only once. Injection was used because *T. castaneum* has been resistant to oral application of dsRNA in our hands. This primary screen revealed 905 *target genes* (5.8% of all protein coding genes) that led to 90% or more lethality. Based on this comprehensive and comparable dataset, GO and KEGG analyses were performed to reveal the biological processes that were overrepresented in this target gene set. A validation screen using lower concentrations of dsRNA and scoring death several times after injection led to the definition of 192 *most effective target genes* that led to death in *T. castaneum* most quickly and at lowest doses. Finally, we tested in how far these target genes could be transferred from injection in *T. castaneum* to oral application in other pest species. Testing 66 genes out of the 192 resulted in 34 *superior target genes* that showed high efficacy after feeding dsRNAs to the mustard leaf beetle *Phaedon cochleariae*.

The genome‐wide screen in *T. castaneum* revealed that good target genes were predominantly involved in basic cellular processes (Buer et al., 2024). The top KEGG and GO terms enriched in the good target genes included transcription, translation, oxidative phosphorylation, proteasome, and phagosome (Figure 1b). Hence, inhibition of basic homeostasis‐related processes rather than very specialized functions turns out to be a more promising RNAi mode of action for pest control. Basic cellular processes were also identified to be effective RNAi targets in other pests. For instance, targeting the proteasome or translations caused high and quick mortality in addition to feeding inhibition in the cabbage stem flea beetle *Psylliodes chrysocephala* (Cedden et al., 2025). Similarly, several proteasome‐related targets achieved feeding inhibition and substantial mortality in *P. cochleariae* and *L. decemlineata* (Mehlhorn et al., 2021; Rodrigues et al., 2021). Of note, our unbiased approach would have had the power to reveal target genes among the class of species‐specific (or lineage‐restricted) genes. However, the majority of the good target genes (90.4%, 818 out of 905, according to ibeetle‐base.uni‐goettingen.de) had other insect orthologs, suggesting there are not many species‐specific effective target genes (Buer et al., 2024). This indicates that evolutionary novel genes may be comparably poor targets potentially due to their less essential functions (Chen et al., 2012).

It is known that different fragments have different RNAi efficiencies, which impacts the lethality read out. However, large‐scale unbiased screening has the disadvantage that it is unrealistic to test two or more fragments per gene because of the scale and the cost of doubling thousands of experiments. As consequence, one might overlook a very effective target gene because the actual region targeted was suboptimal. We suggest using such efficacy prediction to reduce the false negatives in large‐scale approaches and indeed, Buer et al. standardized the selection of the gene region to be targeted using the DEQOR software, which minimizes off‐targets and predicts efficacy based on human siRNA data (Buer et al., 2024) (http://deqor.mpi-cbg.de, unfortunately it is now a proprietary software no longer publicly available since 2023). However, once a small selection of efficient targets is identified, several dsRNA against the same target gene should be designed to identify the best region.

## NO SUCH THING AS ‘THE ONE BEST TARGET GENE’

It has been noted that a gene that is a very effective RNAi target in one species might be comparably ineffective in another species (Mehlhorn et al., 2021). So far, the most systematic demonstration of this was the random selection of 88 genes that showed good dose–responses in the genome wide screen in *T. castaneum* and testing their orthologs in the mustard beetle *P. cochleariae*. Even though both are beetle species, RNAi against only 39 genes (44%) caused lethality above 50% in the second species. Interestingly, the cluster with the best dose response in *T. castaneum* (high lethality even at 3 ng dsRNA injection) also had the highest transferability to *P. cochleariae*: The transferability was 52% for those genes, while it was 22% when the genes were selected from clusters showing less optimal dose responses. In subsequent transfer experiments, the success of transfer was high (above 80%) when the genes first transferred to *P. cochleariae* by feeding were tested in two other leaf beetles (Chrysomelidae) species by feeding (Figure 1a). In the Colorado potato beetle, *Leptinotarsa decemlineata* (Buer et al., 2024), 11 out of 12 tested genes, and in *P. chrysocephala* (Cedden et al., 2025), 11 out of 14 genes from the superior target genes were able to achieve at least 50% mortality.

We suggest that there are several reasons that influence transferability in a species‐specific way. First, gene function and regulation evolve such that the species' reaction to a knock‐down and its propensity for compensation of a knock‐down change. In that respect, it is important to note that all three beetles used for transferring experiments were from the Chrysomelidae (leaf beetles) family, i.e. rather closely related species. The transferability to more distant pests might be lower. Second, the method of delivery might favour certain targets. RNAi treatment in *T. castaneum* was via injection, while it was through oral delivery in the other species. While the gut is clearly the prime target organ after oral delivery, all organs are equally accessible upon injection. Hence, genes essential e.g., in the fat body are well‐targeted after injection but require an additional passage through the gut into other tissues after feeding. This would at the same time explain the comparably poor transferability from our screen to another species by another mode of delivery but the high degree of transferability from oral application in one species to another.

Future studies should test the ‘superior target gene list’ from Buer et al. in other important pest orders such as Hemiptera and spider mites to gain a better understanding of the limits of transferability. It is reasonable to expect that there are overlapping but distinct sets of best target genes in each species, and these gene sets will be more dissimilar between evolutionarily more distant species.

In summary, it is reasonable to assume that there is no such thing as the one best target gene for all species. Rather, past work has defined a pool of potential target genes that differ with respect to parameters including efficacy, transferability across species, and off‐target effects. Large‐scale screens are economically or practically not feasible in most pest species. Hence, in the following section, we suggest using the available information on known effective and highly transferable target genes to screen a handful or some dozens of target genes. This strategy should allow the development of commercial RNAi products for the effective management of important pests if they are susceptible to RNAi in the first place.

## PRACTICAL ADVICE: HOW TO IDENTIFY THE BEST TARGET GENE?

Identifying effective target genes is a crucial step for RNAi‐based management of pest species. Here, we provide recommendations based on our experience with multiple beetle pest species. We recommend testing the orthologs of effective target genes that have been successfully transferred to at least one other pest through oral delivery. Hence, the superior target genes consisting of 34 genes that were successfully transferred to *P. cochleariae* is a great starting point and proved to have ~80% success rate in identifying effective target genes in two other beetle pests (Buer et al., 2024; Cedden et al., 2025). In Table 3, we provide an updated list of effective and transferrable RNAi target genes that is based on successful transfer of effective targets identified in *T. castaneum* to *P. cochleariae*, *L. decemlineata* (Buer et al., 2024), *P. chrysocephala* (Cedden et al., 2025), and western corn rootworm *Diabrotica virgifera virgifera* (Knorr et al., 2018) via oral dsRNA delivery (Table 3). We list in how many pests the orthologs of the respective gene was an effective target, offering a prioritized list of target genes that can be adapted based on the scale of the screen to be conducted in the pest of interest. If testing even more candidates is possible in the pest of interest, the remaining cluster 1 genes from the *T. castaneum* screen (i.e. those that were not tried for transfer to other pests) may be included (see Buer et al., 2024).

**TABLE 3:** List of effective and transferrable RNAi target genes.

*T. castaneum* gene ID^a^	Annotation	Number of transfers
*TC006375*	26S proteasome non‐ATPase regulatory subunit 6‐like protein	4
*TC011120*	Ras opposite	3
*TC005185*	Polyadenylate‐binding protein‐like protein	3
*TC008617*	Proteasome subunit beta type‐4‐like protein	3
*TC010321*	26S protease regulatory subunit 8‐like protein	2
*TC000069*	Proteasome subunit beta type‐1‐like protein	2
*TC002574*	Signal recognition particle 54 kDa protein‐like protein	2
*TC007999*	26S protease regulatory subunit 4‐like protein	2
*TC014725*	Prolactin regulatory element‐binding protein‐like protein	2
*TC015539*	40S ribosomal protein S3a‐like protein	2
*TC015014*	Clathrin heavy chain‐like protein	2
*TC009675*	26S protease regulatory subunit 4‐like protein	2
*TC006492*	26S protease regulatory subunit 8‐like protein	2
*TC004425*	Heat shock 70 kDa protein cognate 3‐like protein	2
*TC012303*	Eukaryotic translation initiation factor 3 subunit A‐like protein	2
*TC000641*	Coatomer subunit beta‐like protein	2
*TC011058*	Dynamin‐like protein	2
*TC033036*	26S proteasome non‐ATPase regulatory subunit 1	2
*TC034312*	Splicing factor 3B subunit 1	2
*TC010318*	Bifunctional 3′‐phosphoadenosine 5′‐phosphosulfate synthase‐like protein	1
*TC010519*	ATP‐binding cassette sub‐family E member 1‐like protein	1
*TC004760*	Hypothetical protein	1
*TC003747*	SAP30‐binding protein‐like protein	1
*TC007324*	60S ribosomal protein L10a‐2‐like protein	1
*TC007891*	26S proteasome non‐ATPase regulatory subunit 8‐like protein	1
*TC013782*	Glycine—tRNA ligase‐like protein	1
*TC014109*	DNA‐directed RNA polymerase II subunit RPB7‐like protein	1
*TC014294*	FACT complex subunit spt16‐like protein	1
*TC014413*	ATP‐dependent RNA helicase WM6‐like protein	1
*TC014785*	Pre‐mRNA‐splicing factor CWC22 homologue‐like protein	1
*TC015205*	Proteasome subunit beta type‐3‐like protein	1
*TC009491*	Putative RNA‐binding protein 15‐like protein	1
*TC009191*	Protein transport protein Sec23A‐like protein	1
*TC008662*	Deoxyuridine 5′‐triphosphate nucleotidohydrolase‐like protein	1
*TC009965*	Ras‐related protein Rab‐3‐like protein	1
*TC010003*	RNA polymerase II, I and III subunit E	1
*TC005653*	Snakeskin	1
*TC005792*	60S ribosomal protein L11‐like protein	1
*TC012381*	Corepressor interacting with RBPJ 1‐like protein	1
*TC000614*	Proteasome subunit alpha type‐6‐like protein	1
*TC011182*	60S ribosomal protein L7a‐like protein	1
*TC008263*	Kinesin‐like protein KLP61F	1
*TC034766*	Tubulin beta‐3 chain	1
*TC031132*	Pre‐mRNA‐splicing factor SYF1	1
*TC011771*	RNA polymerase II 140kD subunit	1
*TC031972*	U5 small nuclear ribonucleoprotein 200 kDa helicase	1

^a^ Details regarding the *T. castaneum* orthologs can be found on ibeetle‐base.uni‐goettingen.de by searching for the gene ID.

We recommend basing the identification of target genes on a thorough orthology inference based on calculating a phylogenetic tree based on protein sequences from *T. castaneum* and the species of interest. The commonly used method based on one‐way BLAST of nucleic acid sequences falls short in two respects. First, the phylogenetic reconstruction is more accurate in identifying the correct orthologs (Emms & Kelly, 2019), and second, it also has the advantage of identifying potential paralogs of the candidates in the species of interest. We recommend removing the candidate genes who have one or more paralogs because these might compensate for the knock‐down (Figure 1c) (Chen et al., 2012). The orthology inference can be conducted using bioinformatic tools such as OrthoFinder (Emms & Kelly, 2019, see Cedden et al., 2025, this issue for example) or retrieved from orthology databases such as eggNOG (Huerta‐Cepas et al., 2019) and OrthoDB (Kuznetsov et al., 2023), if the species of interest is included. MEGA is a free software with extensive functionality for phylogenetic sequence analysis (Tamura et al., 2021).

Screen‐based approaches often identify multiple promising target genes with similarly high lethal effects (Buer et al., 2024; Mehlhorn et al., 2021). For prioritization, we suggest assessing a range of additional important parameters including time until the lethal effect is observed, dose–response curves and reduction in crop damage prior to death (i.e. measuring the consumption of crop after treatment). In addition, in silico prediction of off‐target sequences that would hit essential genes in non‐target organisms will influence the selection of the sequences to be used for application (Cedden et al., 2025; Mogren & Lundgren, 2017). For instance, Cedden et al. (2025) investigated potential off‐targets in five non‐target organisms of effective target gene sequences in *P. chrysocephala*. They found that designing an off‐target minimized dsRNA against *Pchr‐rpt4* was very difficult while it was possible to design such an off‐target minimized dsRNA against *Pchr‐rpt1*. Both genes had shown very similar efficacy in controlling *P. chrysocephala*. Applying these criteria, the number of potential target sequences can be reduced in a rational way.

## WORD OF WARNING: LETHALITY IS AN UNSPECIFIC READOUT THAT MAY LEAD TO FALSE POSITIVE RESULTS

The ultimate aim is the induction of lethality in pest species. Therefore, we recommend basing the target gene assessment on this phenotypic readout. However, lethality is also a very unspecific phenotype that can occur by a non‐optimally broken or contaminated injection needles, contamination of the dsRNA preparation, by the infection status of the animals and many other experimental issues. Mehlhorn et al. (2021) therefore recommend that the efficacy of RNAi upon feeding is first tested using genes that lead to specific morphological phenotypes such as the inhibition of cuticle tanning via the RNAi of *laccase* or developmental genes (Arakane et al., 2005; Mehlhorn et al., 2021). Further, apparently positive results need to be confirmed by a biological replicate starting from an independent dsRNA preparation. Compared to a phenotypic readout, we feel that measuring the reduction of expression via qPCR has a rather limited value. First, it is not known, what degree of knock‐down on the mRNA level is required to induce a phenotypic effect. Second, the complex gene regulatory compensation mechanisms can even lead to apparent lack of downregulation while a clear phenotypic effect is seen (see Kaufholz et al., 2024 for an example). Third, the timing of qPCR after injection may influence the degree of knock‐down that can be observed (e.g. Willow et al., 2021, see Mehlhorn et al., 2021 for further discussion). All this variability reduces the value of qPCR for confirming efficient knock‐down.

## OUTLOOK

The partial overlap of our genome‐wide view with some of the best target genes identified in previous work indicates that the quest for the best target genes has reached some saturation. The financial effort and the experimental challenges of genome‐wide screens are considerable. Therefore, we expect not too many additional large‐scale efforts. Rather, the more focused approach suggested above is likely to reveal some of the best target genes for most pest species with reasonable effort. Still, some additional unbiased screening might be valuable for clades that are economically important but phylogenetically more distant to holometabolous insects such as spider mites. Fungi have quite different physiology and, therefore, may contain classes of targets not covered in any effort done in insects.

The predominance of highly conserved target genes involved in very basic cellular processes and the low abundance of species‐specific genes (9.6% of good target genes) in our genome‐wide screen indicate that species‐specific genes may in most cases not be essential enough for pest control. In contrast, it remains an open question, in how far other types of targets (such as non‐coding RNAs) might lead to efficacies comparable or even superior to protein coding genes.

It remains controversial, in how far targeting two genes at the same time may lead to an additive or a synergistic effect. In case of additive effect, any combination of the best target with a somewhat less efficient target would reduce the overall efficacy. Synergistic effects using two target genes was reported e.g. by Wang et al. (2024) in aphids and Kwon et al. (2016) in mites. In contrast, Ulrich et al. (2015) found no synergy by targeting all combinations of two genes from a set of 11 top target genes.

There is growing interest in developing RNAi‐based management strategies that target multiple species at once, which might be economically advantageous to targeting a single pest species in some circumstances (Wang et al., 2021, 2024). These strategies rely on identifying long and conserved transcript regions across several species to target all of them at once with a single dsRNA. The expansion of lists of effective target genes in different pests might be necessary to achieve the potential of this strategy because the more species should be targeted, the less likely it becomes to find dsRNAs targeting several species. Hence, many potential transcript sequences might have to be screened to find a suitable sequence for the targeting multiple pests.

Another open question relates to the design of the dsRNA sequences. Past efforts in insects have mainly focused on testing long dsRNAs representing continuous stretches of target gene mRNA sequences. Much less effort has been put into the rational design of the dsRNA sequence itself taking the individual efficacy of siRNAs into account. Hence, a further increase in efficacy might be possible by optimizing the sequences.

## AUTHOR CONTRIBUTIONS

**Doga Cedden:** Conceptualization; writing – original draft; writing – review and editing; visualization. **Gregor Bucher:** Conceptualization; writing – original draft; writing – review and editing; supervision.

## CONFLICT OF INTEREST STATEMENT

The authors have no relevant financial or non‐financial interests to disclose.

## Data Availability

Data sharing is not applicable to this article as no new data were created or analyzed in this study.
